# Psychological well-being, food insecurity, academic performance and other risk factors in a sample of university students in Jordan during COVID-19

**DOI:** 10.1017/jns.2024.67

**Published:** 2024-10-10

**Authors:** Tamara Y. Mousa, Latefa A. Dardas

**Affiliations:** 1 Department of Nutrition and Food Technology, School of Agriculture, The University of Jordan, Amman, Jordan; 2 Department of Community Health Nursing, School of Nursing, The University of Jordan, Amman, Jordan

**Keywords:** Arabic psychological general well-being index-short version (PGWB-S), COVID-19, Food insecurity, Jordan

## Abstract

This research validated an Arabic version of the Psychological General Well-being Index-Short version (PGWB-S) and examined the relationship between perceived psychological well-being, and food insecurity, academic achievement, and other risk factors in a sample of university students in Amman, Jordan, during COVID-19. A cross-sectional study was conducted in two phases. Phase 1 translated and validated the Arabic copy of the PGWB-S in 122 students from the University of Jordan. In Phase 2, 414 students completed the demographic questionnaire, Arabic versions of the PGWB-S, the Ryff Psychological Well-being Scale, and the Individual Food Insecurity Experience Scale. The participants had a mean PGWB-S score of 15.82 ± 0.34, and 41.3% had a mean score below 15. Psychological well-being was better in students younger than 21 and/or who had a GPA ≥3.0, were of normal weight or overweight, physically inactive, and food secure, did not drink coffee or smoke, as well as in those whose neighbourhood contained grocery stores and/or public transportation (P < 0.05). In conclusion, during the pandemic, perceived mental well-being was moderate in a Jordanian sample of university students. Perceived psychological well-being was also positively associated with food security and academic performance. These findings suggest that improving food security and academic achievement may contribute to enhanced psychological well-being among university students. Therefore, higher education institutions with the help of the government are encouraged to facilitate the provision of mental health care services to students, mainly post the coronavirus, which according to our knowledge is limited.

## Introduction

Jordan is a developing Arabic country that, since the millennium and the COVID-19 pandemic, has been exposed to socio-economical and medical changes.^(1)^ This is characterized by unhealthy eating behaviours and the development of chronic diseases. It has been found that 25% of Jordanians suffer from hypertension, 20% have cardiovascular diseases, and 18% experience depression.^(2)^ A recent survey reported that 38.4% of 5,274 Jordanians had symptoms of anxiety in the last week of March 2020, which was the onset of COVID-19.^(3)^ In fact, the first case was diagnosed on 2 March 2020, and many more cases followed.^(1)^ This state caused the Jordanian Ministry of Health to announce lockdown on March 17, then the curfew on March 20. Consequently, education in schools, colleges, and universities has switched to online learning.^(1,3)^ However, prior to the epidemic, Malak and Khalifeh (2018)^(4)^ observed that 42% of teenage school students (12–18 years) had symptoms of anxiety, and 74% were depressed. Another study noted that 64% of 600 participants suffered from psychological problems such as stress, depression, or anxiety.^(5)^ Conversely, Hamdan-Mansour and Marmash^(6)^ indicated that most of the 1,108 students indicated moderate psychological well-being, which is defined as the ability of individuals to feel good about their lives, cope with stressful situations, as well as be functional and useful to their society.^(7)^ Similar controversies were found in recent investigations among Jordanian high school^(8)^ and university students,^(9)^ as well as Arab university students.^(10)^ Understanding the mental health of university students, which could be compromised due to the spread of the coronavirus, is vital, as this population faces unique stressors and challenges. Yet, none of the previous studies have adequately addressed this health issue in this particular population across Jordanian universities.

Furthermore, the recent pandemic and its effect on food accessibility, availability, and/or security in Jordan^(1)^ would naturally alter the psychological well-being of individuals.^(11–13)^ Food insecurity is described as the lack of nutritional well-being caused by the incapacity to buy healthy, nutritious food in sufficient quantities.^(14)^ This is crucial since 24% of Jordanians are poor,^(1)^ and 17% are food insecure.^(15)^ Additionally, inadequate intakes of nutritious foods weaken physical and mental health,^(12,16)^ as well as academic performance.^(13)^ This is due to deficiencies in iron, calcium, zinc, vitamins B, C, and D, and omega-3 fatty acids.^(16)^ Several studies also support the current rationale that there is a direct association between low psychological (including, perceived anxiety, depression, or stress, and/or self-perceived mental health status) well-being and food insecurity.^(11–13,17–21)^ For instance, lack of food security has increased the risk of psychological distress and average to very poor self-perceived mental health status by 3.6 and 2.7 folds, respectively, in 302 American college students when compared with food secure students (P < 0.05).^(11)^ Moreover, food security and mental health are proposed to influence academic achievement of students (via using GPA as proxy). In line with this, food insecurity was associated with poor psychosocial health (β = 0.22), which in turn was associated with having a lower GPA (β = −0.21) in 2,377 American university students (P < 0.001).^(13)^

In Jordan, there are numerous questionnaires in Arabic that are used to assess perceived psychological well-being in students including Ryff’s Psychological Well-being Scale,^(6)^ Depression, Anxiety, and Stress Scale,^(5,8–10)^ Symptom Checklist-anxiety, and Center for Epidemiological Studies Depression Scale for Children.^(4)^ Nonetheless, the first couple of tools are long and time-consuming,^(5,6,8–10)^ and the latter two are only used in children (<18 years). Consequently, the researchers chose the short version of the Psychological General Well-being Index (PGWB-S), which is used to discern psychosomatic health status in college students, and can be completed promptly short time.^(22)^ Thus, the primary aim of this study is to validate an Arabic version of the PGWB-S, and the second is to examine the relationship between perceived psychological well-being, and food insecurity, academic achievement, and other risk factors in a sample of Jordanian students from a university in Amman, the capital of Jordan, during COVID-19. Moreover, we targeted this age group because approximately a third of the Jordanian population is young (15–29 years), of whom 285,000 are university students.^(1)^

## Methods

### Design and nature of study

The researchers conducted a cross-sectional study in 2 phases to assess perceived psychological well-being and its relationship with food insecurity, grade point average (GPA), and other risk factors in a sample of university students in Amman, Jordan, during COVID-19. Phase 1 was performed in winter 2021 to translate and validate an Arabic copy of the PGWB-S in 200 students from the University of Jordan in Amman. It is the oldest and largest university in Jordan, and its diverse student body represents 17% of total students in the country.^(1)^ In phase 2, which was performed in spring 2022, the investigations explored perceived psychological well-being, using the validated Arabic Version of the PGWB-S, and its relationship with food insecurity and other risk factors in 470 students. Participants of both phases received an email requesting that they join the study. The email also described the nature and risks of the research, and followed by a link that directed students to a secure research-based website. All participants provided written informed consent via email. The Institutional Review Board at the University of Jordan, Amman, Jordan, approved the protocol of this research.

### Participants

Power analysis showed that a one-tailed *t*-test required a sample size of 111 students for phase 1, and 240 individuals for phase 2 to yield 95% power. The number of students attending the University of Jordan in 2021/2022 was around 47,000.^(1)^ Thus, considering a response rate of ∼50%, random samples of 200 students for phase 1, and 470 students for phase 2 from all the faculties of the University of Jordan were contacted by email with the coordination of the IT department. Students were excluded from participating in this study if they were not Jordanians, younger than 18 years, or sporadically registered in the university (for example; enrolled for a single semester). The response rate was 88.1%, thus 414 students participated in this research.

### Procedure

The PGWB-S^(22–24)^ was translated into Arabic by an expert in the terminology covered by the scale. Next, a panel of bilingual health professionals reviewed the translation. This was followed by back-translation to English by another bilingual health professional and a professor of English literature whose mother tongue is English, to ensure the quality of the translation. This procedure followed the standardized translation method described by the World Health Organization (WHO).^(25)^ Content validity was verified by a panel of five experts in mental health from the departments of Community Health Nursing and Educational Psychology at the University of Jordan. This panel also evaluated the content, readability, difficulty, and bias of the scale items. Furthermore, a focus group of 15 students was selected randomly from the university student lists based on their student ID numbers. These students were contacted by email, but only ten students participated. The focus group estimated the readability and clarity of the scale items via highlighting any word or statement that they found difficult to understand, and thus required clarification. Subsequently, the qualitative input of the expert panel and focus group was incorporated into the final version of the scale.

Following the same approach and the exclusion criteria mentioned above, 200 students were contacted by email. A total sample of 122 participants completed the Arabic version of the PGWB-S twice, over a period of one week. The collected data then was used in the psychometric analyses of the PGWB-S. This analysis showed that the Arabic copy of the PGWB-S had almost perfect agreement via having Cohen’s Kappa of 0.95.^(26)^

### Ethical approval and consent to participate

This manuscript has been read and approved by all authors. The authors confirm that there are no other persons, who satisfied the criteria for authorship, but are not listed. The order of authors listed in the manuscript has been approved by all of them. They also understand that the Corresponding Author is the sole contact for the Editorial process, and holds the responsibility for communicating with the other author about progress, submissions of revisions and final approval of proofs. Moreover, the authors declare that this manuscript is original, has not been published before, and is not currently being considered for publication elsewhere. We confirm that there are no known conflicts of interest associated with this publication, which did not receive any financial support. Finally, the reporting of this work is compliant with The Code of Ethics of the World Medical Association (Declaration of Helsinki). In addition to that, the protocol of this research is approved by the Institutional Review Board at the University of Jordan, Amman, Jordan (ref no.: 2021-89). Moreover, an informed consent was obtained from all subjects involved in both phases of the study, as well as permission to use the tools of assessment was obtained from all researchers.

### Tools of assessment

**Demographic questionnaire** collected information about age, sex, academic specialization and year, grade point average (GPA), weight and height that are used to calculate body mass index (BMI), health history, physical activity, smoking, and drinking coffee, socio-economic data (including governorate, employment, and family income), and presence of grocery stores and/or transportation system in the neighbourhood.

The PGWB^(23,24)^ and its short version^(22)^ are one of the tools used to identify the psychosomatic health status of the previous month, via its six sub-scales: anxiety, depression, positive well-being, self-control, general health, and vitality. In addition, this study used the PGEB-S due to being timely, as well as used to assess mental well-being in university students.^(27–29)^ The **PGWB-S** is a 6-item questionnaire. Each item has a 6-point Likert scale with a response range of 0–5, which requires 5 minutes to complete. The total score ranges between 0 and 30, where a larger score indicates better psychological well-being.^(22–24)^ Grossi *et al.* (2006)^(22)^ did not define a cut-off point for the total score of PGWB-S. Nevertheless, since the mean score was 15.8 and the data was normally distributed, we considered 15 as the cut-off point. Thus, any participant who scored >15 is considered to have a positive perception of psychological well-being.

**Arabic Version of Ryff Psychological Well-being Scale** is a 54-item questionnaire. This is a 6-point Likert self-administered scale, which has a response range of one (strongly disagree) to six (strongly disagree). The total score ranges between 18 and 108, with a cut-off point of 63. This tool measures six aspects of perceived psychological well-being, including autonomy, positive relations with others, environmental mastery, personal growth, purpose in life, and self-acceptance. This Arabic copy that was tested in 1,101 Jordanian university students had a Cα 0.83.^(6)^

**Individual Food Insecurity Experience Scale** (FIES) is a validated Arabic questionnaire that is used to evaluate the level of food security over a period of four weeks.^(14,30)^ The FIES consists of eight questions asking about food availability at home and having the money to buy food, and it takes 5 minutes to finish. A higher total score, which ranges between zero and eight, reflects lower food security status. Based on the overall score, the degree of lack of food security is categorized into food secure (FIES = zero), and moderately (FIES = 1), or severely food insecure (FIES = 2–3).^(14,30–32)^

### Statistical analysis

Statistical analyses were conducted using the statistical software Graduate Pack IBM SPSS Statistics for Windows version 19.0 (SPSS 19; IBM, 2010). To ensure validity of the entered data, we performed a thorough univariate analysis, and inspected the data for outliers or missing entry. Then we explored the degree of randomness of the missing data points via a multivariate diagnostic test, followed by assigning the missing data using Munro’s methods.^(33)^ Non-directional statistical tests were tested at 0.05-level of significance. Before analyzing the data, assumptions of all tests were examined. Descriptive statistics were displayed as means ± standard error of the mean (SE) and frequency distributions. Analysis of variance (ANOVA) for continuous variables was used to measure differences among participants. Bonferroni post hoc test was used to compare two or more independent samples of equal or different sample sizes. We executed regression models to examine associations of psychological well-being with food insecurity, and demographic indicators (such as age, gender). In relation to the pilot study, test-retest reliability of the Arabic version of the PGWB-S was computed by an insignificant paired *t*-test (P > 0.05). Internal consistency and Intra-rater reliability of the PGWB-S were estimated using Cα ≥0.6 and Cohen’s kappa, respectively. Concurrent validity also was conducted to compare the total score of the Arabic PGWB-S with that of the Arabic version of Ryff’s Scale.^(6)^ Moreover, our Arabic PGWB-S had an acceptable sampling adequacy with KMO = 0.734, and significant sphericity (Bartlett’s test of P < 0.0001).

## Results

The current sample ranged in age from 18 to 38 years, where 71.5% were younger than 21 years. Participants represented the three Jordanian provinces evenly, and 64% of them were women. The majority of the students were single and unemployed; two-thirds had normal weight; half of them had a GPA ≥3; and one-fifth was overweight or obese.

Based on the findings of phase 1, the Arabic PGWB-S was stable over time, as indicated by the paired *t*-test of times one and two that was insignificant (*t* = –1.317, P = 0.19), and had an intra-class correlation coefficient of 0.87 (95% CI (0.84–0.91), P < 0.001). Moreover, this scale was reliable as it had a good internal consistency as reflected by Cronbach’s α (Cα) of 0.87. Thus, the items of the current PGWB-S are correlated with each other, reflecting their ability to measure perceived psychological well-being status. As a result, we concluded that the Arabic version of the PGWB-S is a valid tool to measure psychological well-being in a Jordanian university population, as reflected by its good internal and external reliability. The present Cα for the overall score (∼0.9) was consistent with that of the original scale when conducted in a student population (Cα = 0.94).^(22)^ Our outcome also was in agreement with the internal consistency of the Portuguese copy of the PGWB-S that assessed psychosomatic status in university students during COVID-19 (Cα = 0.86).^(27)^ For additional validation, the Arabic version of Ryff’s Psychological Well-being was administered to the same participants,^(6)^ to test for concurrent validity with the current PGWB-S. The mean score of the students of Ryff’s scale was 62.8. The total score of the Arabic version of the PGWB-S correlated well with that of Ryff’s questionnaire (r = 0.80, P < 0.05).^(6)^

The students had a mean PGWB-S score of 15.82 ± 0.34, reflecting moderate perceived psychological well-being, which ranged between 2 and 29. Approximately, 27% of the participants scored ≥ 21 on the PGWB-S, and 58.7% had a mean score greater than 15, compared with 41.3% attaining a score below 15 (20.69 ± 0.23 vs. 8.9 ± 0.31, F = 976.43, P < 0.001). Table 1 shows the proportion of students scoring on PGWB-S dimensions. Less than a quarter of the participants frequently perceived themselves as suffering from anxiety or low vitality. Furthermore, often the perception of depression, low positive well-being, lethargy, and a lack of self-control was documented in 15% to 26% of the students.

**Table 1.** Proportion of students scoring on PGWB-S^a^ dimensions (n = 414)

#	Question	PGWB-S dimension	Item (score)					
			n (%)^b^					
1	Have you been bothered by nervousness or your “nerves” during the past month?	Anxiety	**Extremely so - to the point where I couldn’t work or take care of things (0)**	**Very much so (1)**	**Quite a bit (2)**	**Some – enough to bother me (3)**	**A little (4)**	**Not at all (5)**
			**60 (14.5)**	35 (8.5)	68 (16.4)	93 (22.5)	114 (27.5)	44 (10.6)
2	How much energy, pep, or vitality did you have or feel during the past month?	Vitality (energy)	**Very full of energy – lots of pep (5)**	**Fairly energetic most of the time (4)**	**My energy level varied quite a bit (4)**	**Generally low in energy or pep (2)**	**Very low in energy or pep most of the time (1)**	**No energy or prep at all - I felt drained (0)**
			20 (4.8)	90 (21.7)	118 (28.5)	100 (24.2)	46 (11.1)	**40 (9.7)**
3	I felt downhearted and blue during the past month.	Depressed mood	**None of the time (5)**	**A little of the time (4)**	**Some of the time (3)**	**A good bit of the time (2)**	**Most of the time (1)**	**All the time (0)**
			68 (16.4)	90 (21.7)	120 (29)	73 (17.6)	40 (9.7)	**23 (5.6)**
4^c^	I was emotionally stable and sure of myself during the past month.	Self-control	**37 (8.9)**	70 (16.9)	67 (16.2)	87 (21.0)	116 (28.0)	37 (8.9)
5^c^	I felt cheerful, lighthearted during the past month.	Positive well-being	**33 (8.0)**	52 (12.6)	112 (27.1)	96 (23.2)	97 (23.4)	24 (5.8)
6	I felt tired, worn out, used up, or exhausted during the past month.	Vitality (lethargy)	16 (3.9)	44 (10.6)	123 (29.7)	133 (32.1)	40 (9.7)	**58 (14.0)**

a PGWB-S: Psychological General Well-being Index-Short version.

b Percentages in bold reflect the number of students with low psychological well-being.

c The scoring for the items 4 and 5 is reversed; all the time = 5, and none of the time = 0.

The PGWB-S mean scores of the students are presented in Table 2. The mean PGWB-S scores were significantly higher among these aged between 18 and 20 years, and who were in their first, second, or fourth year, and had a GPA ≥ 3.0 (P < 0.05). Psychological well-being perception was also significantly better in adults who were of normal weight or overweight and food secure as compared with obese and food insecure ones (P ≤ 0.002). In addition, participants who drank coffee, smoked, were physically active, and lacked a nearby grocery store and/or public transportation system had significantly lower mean PGWB-S scores than non-coffee drinkers, non-smokers, inactive students, and whose neighbourhood contained a grocery store and/or public transportation system (P < 0.05).

**Table 2.** Perceived psychological well-being (PGWB-S^c^) mean scores of the students (n = 414)

Variable	Mean ± SE	F	P-value
Sex		2.37	0.120
Women	15.43 **±** 0.43^a^		
Men	16.52 **±** 0.56^a^		
Age (years)		5.07	**0.025**
18–20	16.38 **±** 0.46^a^		
21–38	14.78 **±** 0.47^b^		
Year of study		7.94	**0.000**
1st	16.05 **±** 0.54^a^		
2nd	17.64 **±** 0.67^a^		
3rd	12.94 **±** 0.74^b^		
4th	15.63 **±** 0.76^a^		
GPA^d^ levels		23.28	**0.000**
3.0–4.0	17.31 **±** 0.44^a^		
2.0–2.99	12.23 **±** 0.74^b^		
1.0–1.99	10.94 **±** 1.06^b^		
Weight status (BMI^e^ in kg/m^2^)		5.03	**0.002**
Underweight (BMI < 18.5 kg/m^2^)	12.96 **±** 1.69 ^a,^^b^		
Normal weight (BMI = 18.5–24.9 kg/m^2^)	16.12 **±** 0.41^a^		
Overweight (BMI = 25–29.9 kg/m^2^)	16.31 **±** 0.81^a^		
Obese (BMI ≥ 30 kg/m^2^)	10.50 **±** 0.13^b^		
Food security (FIES^f^ score)		42.48	**0.000**
Food secure	16.77 **±** 6.32^a^		
Moderate or severe food insecure	11.13 **±** 7.88^b^		
Income (JD)		0.988	0.373
≤ 2000 JD	15.58 **±** 0.40^a^		
2000–4000 JD	17.29 **±** 1.09^a^		
Do not wish to answer	15.99 **±** 0.77^a^		
Drinking coffee		8.22	**0.004**
I drink coffee	15.17 **±** 0.42^a^		
I do not drink coffee	17.27 **±** 0.57^b^		
Smoking		11.02	**0.001**
I smoke cigarettes, e-cigarette, or nargilah	13.23 **±** 0.81^a^		
I do not smoke	16.30 **±** 0.39^b^		
Physical activity		4.81	**0.029**
Active	15.32 **±** 0.40^a^		
Not active	16.93 **±** 0.63^b^		
Presence of a nearby grocery store		36.74	**0.000**
Yes	16.40 **±** 0.33^a^		
No	9.09 **±** 1.43^b^		
Presence of a nearby public transportation		7.32	**0.007**
Yes	16.31 **±** 0.38^a^		
No	14.11 **±** 0.71^b^		

a,b In columns, different superscripts indicate significant differences within categories at P < 0.05.

c PGWB-S: Psychological General Well-being Index-Short version.

d GPA: grade point average.

e BMI = body mass index.

f FIES: Individual Food Insecurity Experience Scale.

Table 3 indicates that PGWB-S had a weak negative association with age (P = 0.004). Psychological well-being perception, however, significantly increased by 3.3-fold, and decreased by almost 2 and 1 fold for every 1-unit change in GPA, daily number of coffee cups, and FIES total score, respectively (P < 0.05). Nonetheless, a small FIES score reflects greater food security; thus, psychological well-being perception is associated positively with food security status. Moreover, having higher BMI and GPA, and being older and/or food insecure significantly increased the risk of negative perception of psychological well-being (P < 0.001). Lastly, there is a trend towards a weak negative association between psychological well-being perception and BMI (P = 0.052).

**Table 3.** Associations of perceived psychological well-being (PGWB-S) with other indicators (n = 414)^a,^ ^b^

Indicator	β^c^	P	OR	95% CI for OR	P
Age (years)	–0.277	**0.004**	–1.203	–1.106, 1.309	**0.000**
BMI (kg/m^2^)^d^	0.159	0.052	1.186	1.088, 1.292	**0.000**
GPA^e^	3.249	**0.000**	0.371	0.252, 0.546	**0.000**
Daily number of cigarettes	0.049	0.355	0.046	–	0.996
Daily number of coffee cups	–1.849	**0.000**	–0.254	–	0.995
Food security (FIES score)^f^	–0.933	**0.000**	–1.671	–1.427, 1.995	**0.000**

a Data is considered statistically significant at P *<* 0.05. The OR increases for every unit increase in the predictor (independent variable). Reference group is high psychological well-being (PGWB-S>15) (OR = 1).

b PGWB-S: Psychological General Well-being Index-Short version.

c Controlled for all variables.

d BMI = body mass index.

e GPA = grade point average.

f FIES: Individual Food Insecurity Experience Scale. A higher score reflects greater food insecurity.

## Discussion

Validation of the Arabic PGWB-S is an essential and novel finding of this study. This short scale was valid, stable over time, and timely, thus retaining a high completion rate. Using such questionnaire will help in conducting future studies to evaluate mental health in students post the pandemic, to prevent its deterioration that may lead to several psychological problems such as depression, anxiety, sleeping disturbances.

In relation to our findings, after almost 2 years of COVID-19, psychological well-being perception was moderate in the current sample. For instance, the current mean PGWB-S score (15.82 **±** 0.34) was lower than that reported in 300 Portuguese college students (µ = 18.9 ± 4.9),^(27)^ and 317 Italian adults (µ = 19.54 ± 4.65).^(28)^ Moreover, less than half of our participants perceived low psychological well-being during the pandemic. In particular, the students perceived experiencing anxiety, lethargy, depression, a negative sense of well-being, and a lack of liveliness and self-control as well. These figures are lower than the data reported in a couple of studies conducted in Jordan during the coronavirus. In the first research, Olaimat and colleagues^(10)^ observed that in 2021, 70% of 2,083 university students (18 – ≥25 years) showed symptoms of anxiety. In the other recent survey,^(8)^ investigators found that depression (81.8%) and nervousness (74.7%) were prevalent in 384 senior high school Jordanian students (mean age = 17.6 ± 0.5 years). Other surveys in Jordan indicated that depression, anxiety, stress, and/or poor sleep were prevalent among 20–70% of college students,^(34–38)^ and men and women aged 18–65 years^(39)^ during the COVID-19 malady. In the same period, 38%–57% of young Jordanians, Iraqis, Emirates, Egyptians, Omanis, and Saudis aged 15–24 years experienced psychological distress.^(9)^ However, more than a decade prior to the pandemic, a descriptive correlation research observed moderate to high levels of mental wellness among 1,101 Jordanian university students using Ryff’s Psychological Well-being Scale.^(6)^ This discrepancy could be attributed to the different time and kind of tools used to examine psychological wellness, as well as in differences in cultures, communities, economies of the population sample, and COVID-19 severity or the procedures enforced by the government to contain it (for example; use of masks, social distancing, or curfew).

On the other hand, a couple of international cross-sectional studies revealed results that are parallel to our outcomes; 47% of 100 Indian^(29)^ and 46.2% of 302 American^(11)^ university students suffered from psychological unwellness. In contrast, 64% and 53% of 681 adult Kiwis,^(40)^ as well as 64% and 57% of 3,097 British men and women (mean age of 44 years)^(41)^ experienced both depression and anxiety, respectively, throughout the epidemic.

In this study, psychological wellness perception was more prevalent in the younger students (18–20 years) than in these older than 20 (P < 0.05). In Jordan, Almomani *et al.* ^(35)^ support the present outcomes, in which anxiety and depression were lesser in students aged 18–20 years. This is in contrast to a previous research that found better mental health levels among Jordanian university students aged ≥24 years.^(6)^ Yet in New Zealand^(40)^ and the United Kingdom,^(41)^ stress and/or anxiety during the coronavirus wave were lower in men and women aged 25 – ≥75 years when compared with those aged 18–24 years. On the other hand, in the US, psychological distress was more common in men and women aged 18–39 than in adults aged ≥40.^(12,17)^ However, a cross-sectional study observed that distress, nervousness, or depression were highly prevalent in college students aged 18–24 when compared with older students (>24 years).^(11)^ We concluded that the relationship between age and psychological well-being varies, which warrants further investigation in the future since many variables (socioeconomic status, pandemics, or wars) could mediate such association.

Mental distress would adversely influence the health status of individuals. This inference is supported by a recent national survey. In 2020, the WHO reported that suffering from stress, anxiety, lethargy, and/or depression is linked to engaging in negative behaviours like smoking, physical inactivity, and/or drinking alcohol, and developing chronic diseases including cardiovascular diseases, respiratory diseases, diabetes mellitus, or cancer.^(2)^ This suggestion raises the alarm regarding the mental health of university students (and probably school students as well). For instance, stressful situations such as the quarantine associated with the COVID-19 era, and other conditions including suffering from verbal, physical, and/or sexual abuse, having a handicap, or bullying would have detrimental effects on the physical, nutritional, and mental health of students, as well as on their academic performance. In line with this, we found that perceived mental well-being was significantly better in students with higher academic achievement, as indicated by GPA, than in these with low academic performance (P < 0.001). Findings of a recent cross-sectional report, which explored depression, anxiety, and stress during COVID-19 in 1,380 undergraduates, agree with the present outcome. For instance, anxiety was less prevalent in students with a very good GPA.^(38)^ Results of Alqudah and others^(36)^ and Raskind *et al.* ^(13)^ were comparable with ours. The authors observed lower levels of anxiety and/or depression among undergraduates with a GPA ranging between good and excellent.^(13,36)^ Therefore, having good school achievement would predispose to having a positive mental status. This is why it is vital to provide counseling to students, especially to these with low grades.

In addition, the current normal-weight or overweight students significantly perceived having a healthier mental status than obese students (P < 0.05). This could be due to that obese individuals suffer from body image or weight dissatisfaction and the desire to lose weight, predisposing them to depression, as stated in numerous studies.^(42,43)^ Unfortunately, body image dissatisfaction was not measured in the present study; hence, it is recommended to assess the role of body and weight dissatisfaction in mediating the relationship between both mental and weight statuses in Jordanians.

The present data showed that psychological well-being perception was better in food secure students by about 2-fold when compared with the food insecure ones (P < 0.0001). In the same stressful year, low food security increased the risk of anxiety by 3.6-fold and depression by 3.5-fold in 2,714 low-income Americans (≥18 years) (P < 0.05).^(12)^ Another national survey reported that food insecurity was high among the 1,476 low-income adult Americans who suffered from mental problems including stress and depression.^(17)^ Comparable findings were seen prior to the pandemic in 18,090 Kiwis aged ≥ 15 years,^(18)^ 426 teenage Spanish boys and girls,^(19)^ and 302^(11)^ and 2,377 American university students.^(11,13)^ To our knowledge, this is the first research documenting that food insecurity would predispose a Jordanian sample of university students to perceive poor psychological well-being during the COVID-19 pandemic. However, large cohorts and national studies must be conducted to confirm this result.

The sample of Jordanian students who drank coffee perceived experiencing psychological illness more frequently than non-coffee drinkers did. Similarly, higher anxiety was observed in 736 Jordanian university students who regularly drank both coffee and tea.^(36)^ Moreover, psychological ill-being was more pronounced in students who smoked. This finding is in parallel with that of Hamaideh *et al.*,^(38)^ and a national survey^(37)^ that showed greater anxiety and nervousness in healthcare students (mean age is 21.62 ± 4.9 years) who smoked. Interestingly, Alqudah and colleagues^(36)^ found that anxiety was worse in negative smokers (µ = 26.41 ± 11.41) as compared with smokers (µ = 22.94 ± 11.68) and non-smokers (µ = 22.25 ± 11.36). It is suggested that distressed, depressed, or nervous individuals would smoke^(44)^ or drink coffee^(45,46)^ to relieve stress. Nevertheless, future studies should examine this proposition, as well as explore the association between mental wellness and negative smoking.

In the present study, psychological well-being perception was lower in students who exercised. In contrast, Guidetti and others^(32)^ noticed that the highly active men and women had a significantly greater mean PGWB-S score than these of low physical activity (20.07 ± 4.63 vs. 18.57 ± 4.08, P < 0.05). This indicates that engagement in physical activity would improve mental health during quarantine,^(32,35)^ which disagrees with the present findings. This might be due to that students who exercise do not have a lot of time throughout the day to do so, which exerts pressure on them to maintain such a healthy, active lifestyle. However, additional investigation in this area is needed to explain such contradictory findings.

Finally, we noticed that perceived mental and emotional wellness was poorer in the students who lacked a grocery store, and/or public transportation system in their neighbourhood. This novel finding could be explained by that food and transportation accessibility could minimize stress via facilitating the purchase of food or reaching destinations such as the university. Yet, prospective studies should examine such interpretations.

Limitations of this research are the cross-sectional design that prevented assessment of causality and generalizability of the present findings, using the mean as the cut-off point, and the shortage of funding that prevented applying a clinical interview for the participants who had low scores (<15). Nonetheless, the present study had numerous strengths. First, a large sample was used; second, the population sample included men and women from different specializations, socioeconomic statuses, and geographical areas of the country; and third, the translation procedure. Finally, the current research is the primary one to measure the association between psychological well-being perception and food security in a sample of Jordanian university students, as well as to translate and validate an Arabic version of the PGWB-S. Lastly, this investigation documented the relative validity of the translated PGWB-S by having a significant correlation with Ryff’s Psychological Well-being Scale.

## Conclusions

During the pandemic, perceived mental well-being was moderate in a Jordanian sample of university students. The current study also documented that the perception of mental wellness was more prevalent in students younger than 21, who had a GPA ≥ 3.0, and/or were of normal weight or overweight, physically inactive, and/or were food secure. Furthermore, perceived psychological health was better in the students who did not drink coffee or smoke, were food secure, and whose neighbourhood contained grocery stores and/or public transportation. These findings suggest that targeted mental health care interventions, including support for food security and academic success, can potentially improve students’ overall well-being. These findings would help researchers in developing interventions to detect problems in nutritional status and mental health of university students, and their response to traumas post the COVID-19 pandemic, such as losing loved ones, bullying, abuse, inaccessibility to food markets, or the pressure of academic success. This is vital to prevent food insecurity, and deterioration of both mental and physical health that could predispose individuals to malnutrition, chronic diseases, distress, addiction, or even ending their life. Therefore, higher education institutions with the help of the government are encouraged to facilitate the provision of mental health care services to students, mainly post the coronavirus, which according to our knowledge is limited. Finally, future longitudinal studies are warranted to understand the magnitude of mental wellness in different universities and socio-cultural areas of Jordan.

## Abbreviations

**PGWB-S:** Psychological General Well-being Index-Short version; **GPA:** grade point average; **BMI:** body mass index; **FIES:** Individual Food Insecurity Experience Scale.

## Conflict of interest

There was no conflict of interest.

## Funding received

This research did not receive any specific grant from funding agencies in the public, commercial, or not-for-profit sectors.

## Authorship contributions

**T.Y. Mousa** designed the research and back translated the Arabic version of Psychological General Well-being Index-short version (PGWB-S) to Arabic. She also contacted and communicated with the panel of experts, as well as with the focus group. Accordingly, she incorporated the qualitative inputs of the panel groups and focus groups into the final version of the PGWB-S. In addition, she communicated with the IT department to send the surveys to the students. Finally, she conducted statistical analysis and wrote the manuscript.

**L.A. Dardas** translated the PGWB-S to English, as well as contacted and communicated with the panel of experts. She also incorporated the qualitative inputs of the panel groups and focus groups into the final version of the translated PGWB-S, conducted psychometric and statistical analyses, and contributed to manuscript writing.

## Availability of data and materials

**Title**: Psychological Well-being, Food Insecurity, Academic Performance and Other Risk Factors in a Sample of College Students in Jordan during COVID-19

**Repository type**: [Data set]. Zenodo.

**DOI**: https://doi.org/10.5281/zenodo.7636433

**License**: Creative Commons Attribution 4.0 International

**Publication date:** February 13, 2023

**Description**: This research investigated psychological well-being perception and its relationship with food insecurity, academic performance and other risk factors in a sample of university students in Amman, Jordan during COVID-19. A cross-sectional study was conducted in two phases. Phase 1 translated and validated an Arabic version of the Psychological General Well-being Index-Short version (PGWB-S) in 122 university students. In Phase 2, 414 students completed demographic questionnaire, Arabic Versions of the PGWB-S, Ryff Psychological Well-being Scale, and Individual Food Insecurity Experience Scale.

**Ethical considerations**: This manuscript has been read and approved by all authors. The authors confirm that there are no other persons, who satisfied the criteria for authorship, but are not listed. The order of authors listed in the manuscript has been approved by all of them. They also understand that the Corresponding Author is the sole contact for the Editorial process, and holds the responsibility for communicating with the other author about progress, submissions of revisions and final approval of proofs. Moreover, the authors declare that this manuscript is original, has not been published before, and is not currently being considered for publication elsewhere. Furthermore, all data used in the study is confidential and the lead author has full access to the data reported in the manuscript. We confirm that there are no known conflicts of interest associated with this publication, which did not receive any financial support. Finally, the reporting of this work is compliant with The Code of Ethics of the World Medical Association (Declaration of Helsinki). In addition to that, the protocol of this research is approved by the Institutional Review Board at the University of Jordan, Amman, Jordan (ref no.: 2021-89).

## Reporting guidelines

This article followed the STROBE checklist.
